# Optimization of instrument conditions for the analysis for mercury, arsenic, antimony and selenium by atomic absorption spectroscopy

**DOI:** 10.1016/j.mex.2018.07.016

**Published:** 2018-07-29

**Authors:** Elisabeth Mohammed, Terry Mohammed, Azad Mohammed

**Affiliations:** a Department of Chemistry, The University of the West Indies, St Augustine, Trinidad and Tobago; b Department of Life Sciences, The University of the West Indies, St Augustine, Trinidad and Tobago

**Keywords:** Hydride generation atomic absorption spectrophotometry (HGAAS), Cold vapor atomic absorption spectrophotometry (CVAAS), Mercury, Arsenic, Antimony, Selenium VGA77, HGAAS, CVAAS

## Abstract

The chemical vapor generation atomic absorption spectrometry technique is extremely popular for trace analysis specifically hydride generation continuous flow systems for arsenic, antimony, selenium and cold vapor for mercury. Optimizing the instrument parameters as well as the hydride generating reactions will improve the sensitivity and reliability of the results obtained. The advantage of optimizing these conditions increases the production of hydrides or vapor species formed thereby improving recoveries. In addition this helps to reduce chemical interferences from other species that may compete with the analyte of interest for hydride formation. Parameters optimized include:

• Reagent flow rate
• Sample flow rate
• Argon flow rate
• Acetylene/Air ratio
• Concentration of reagents
• Read delay time

For the analytical procedure the flow rate of the reagents and sample was affected by the tension on the peristaltic pump and the size of the tubing. The optimized flow rate for all reagents was between 0.9–1.0 mL/min and between 6–7 mL/min for the sample when both conditions were applied. The optimized type and concentrations of the reducing agent for Arsenic, Antimony and Selenium were NaBH4 (0.6% w/v), NaBH4 (0.7% w/v) and NaBH4 (0.1% w/v) in NaOH (0.5% w/v) respectively and SnCl2 (25% w/v) in HCl (20% v/v) for Mercury. The concentration and type of acid that produced the optimum signals for Arsenic, Antimony and Selenium were 5, 10 and 10 mol/dm-3 respectively. The flow rates for the carrier gas (Argon) for Arsenic, Antimony, Selenium and Mercury were optimized at 0.2, 0.2, 2.0 and 2.0 mL/min respectively. The optimized flow rate for fuel gas (Acetylene) for all the metals except Mercury was 2.5 mL/min. The optimized Instrument Read Delay Time for Mercury was 70 s and 20 s for Arsenic, Antimony and Selenium.

## Graphical abstract

**Figure:**
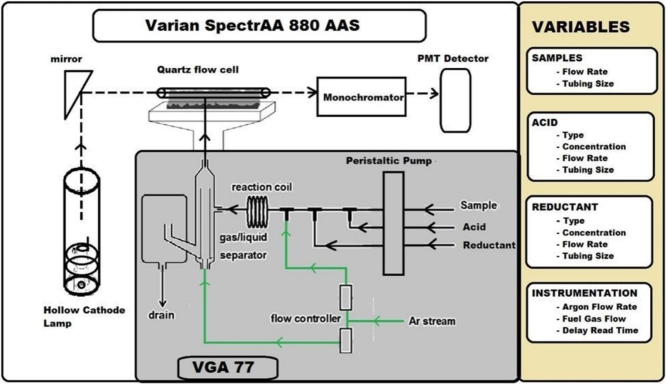

**Specifications Table**

**Table:** 

Subject area	*Select one of the following subject areas:* • *Chemistry*
More specific subject area	*Analytical Chemistry*
Method name	*Hydride Generation Atomic Absorption Spectrophotometry (HGAAS), Cold Vapor Atomic Absorption Spectrophotometry (CVAAS)*
Name and reference of original method	*NA.*
Resource availability	*NA*

## Method details method

Chemical vapor generation in conjunction with atomic absorption spectroscopy is one of the most powerful tools for the determination of trace elements in a variety of matrices. Chemical vapor generation techniques include hydride generation atomic absorption spectroscopy (HGAAS) and cold vapor atomic absorption spectroscopy (CV-AAS).

The hydride generation technique makes use of the separation of the analyte from the matrix by conversion to the volatile hydrides and offers a pathway to trace analysis of elements such as As, Sb and Se that cannot be determined by conventional methods. Excellent low levels of detection are attained when this separation method is combined with atomization of the hydride in a heated quartz tube. The sensitivity of this technique is due to its ability to determine the levels of these trace metals at wavelengths below 200 nm, a region where there is considerable spectral interferences from radicals in conventional flame AAS [1]. The preparation of the sample will receive little attention in this paper but it must be noted that no detection technique will produce accurate results unless the sample preparation (digestion procedure) quantitatively releases the bound analyte element and converts it to its appropriate valency state.

The HG-AAS technique involves three distinct processes [2]:

1 Hydride generation and release of the element of interest from the sample solution (sample converted to hydride transferred to gaseous phase) with the aid of a reducing agent.
2 Hydride collection (optional)
3 Atomization- transport of the released hydride to the atomizer by the flow of an inert purge gas.

The factors that will influence the yield of the hydrides and the sensitivity of the technique include:

1 The concentration of the acid
2 The concentration and type of the reducing agent (the facile production of hydrides by reaction with reducing agent)
3 The flow rate of the carrier gas
4 Flow rate of the reducing agent
5 Flow rate of the acid
6 The read delay time of the sample

Mercury determination through flame atomic absorption spectroscopy (FAAS) gives rise to poor sensitivity [3]. An alternative absorption technique such as cold vapor atomic absorption spectroscopy (CVAAS) is one of the most sensitive method for mercury determination. The principle of this technique is as follows:

1 A reducing agent typically stannous chloride (SnCl_2_) is used to convert ionic metal ions of Mercury^2+^ (aq) in solution to Hg^0^ (g) which produces a higher analytical absorbance signal.
2 Mercury having a high vapor pressure is liberated and entrained in an inert gas of Argon after passing the liquid gas separator.
3 The Mercury vapor is transferred into the optical path of the atomic absorption spectrophotometer where Mercury absorbs light of a specific wavelength at room temperature.

Therefore the sensitivity and precision can be maximized by optimizing the parameters essential for increasing the supply rate of gaseous mercury and consequently the analytical signal depends on the transport efficiency of the gaseous chemical species (elemental Mercury). These include:

1 Concentration of the reducing agent
2 Flow rate of reducing agent (SnCl_2_)
3 Flow rate of the sample
4 Flow rate of the purge gas
5 Read delay time

## Experimental

### Instrumentation and materials

• Varian Atomic Absorption Spectrophotometer (SpectrAA880)
• Agilent-VGA 77 accessory
• Inorganic ventures stock solutions of Mercury (1000 mg L^−1^), Arsenic (1000 mg L^−1^), Antimony (1000 mg L^−1^) and Selenium (1000 mg L^−1^)
• Sigma Aldrich Tin (II) Chloride (reagent grade, 98%)
• Sigma Aldrich Sodium Borohydride (ACS grade)
• Sigma Aldrich Sodium Hydroxide (ACS grade)
• Scharlau Hydrochloric Acid (ACS grade)
• Sigma Aldrich Potassium Iodide (ACS grade)
• Agilent mercury flow through cell
• Agilent quartz flow through cell
• Gilson Medical Electronics silicone tubing, internal diameters: 0.63, 0.67 and 0.76 mm (Reagent)
• Gilson Medical Electronics silicone tubing, internal diameters 2.28, 2.54 and 2.92 mm (Sample)
• Stop watch
• Measuring cylinders (25 mL)

**Fig. 1:**
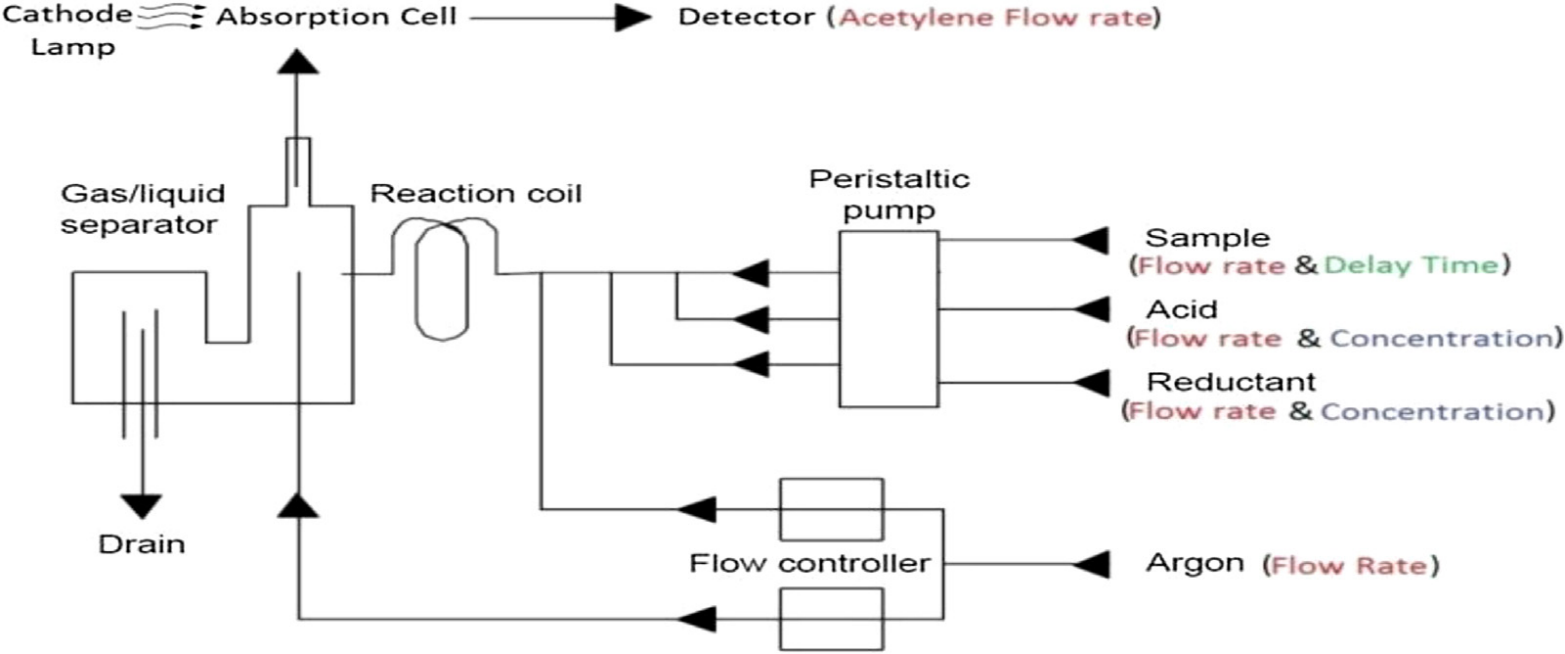
Schematic of the VGA accessory coupled to the atomic absorption spectrophotometer and the optimization parameters for HGAAS and CVAAS.

Calibration standards of 10, 30 and 50 ppb were prepared for each metal being analyzed in 50 mL volumetric flasks. The 30 ppb standard of each metal was used for optimization of the instrument parameters. Fig. 1. Shows a schematic illustrating the parameters were optimized for both HGAAS and CV-AAS. Acetylene flow rate and acid concentration were two additional parameters that were optimized for HG-AAS. Mercury was optimized first since the Potassium Iodide used to reduce As, Sb and Se has a negative interference on Mercury.

Table1. Shows the range selected for each parameter optimized. The varying concentrations of reducing agent and acid solutions were prepared in volumetric flasks by weighing the appropriate masses of the reagent and then diluting to 250 mL with deionized water. To determine the optimized concentration for the reducing agent and acid, a 30 ppb standard was used. The AAS was zeroed after each modification and absorbance were measured for each variation in solution. Deionized water was pumped through the entire system for 60 s between analyses to prevent cross-contamination.

**Table 1:** Range of each parameter used for the optimization of the instrument.

Parameters	Arsenic	Antimony	Selenium	Mercury
Reducing Agent type	NaBH_4_ in NaOH			SnCl_2_ in HCl
Reducing agent concentration (%)	0.1, 0.4, 0.6 and 0.7 % (w/v) NaBH_4_ in 0.5% (w/v) NaOH			15, 20, 25 and 35% (w/v) SnCl_2_ in 20% (v/v) HCl
Acid type	HCl			Deionized water
Acid concentration (M)	3, 5, 7, 10 and 12			NA
Flow rate of reducing agent and acid (mL/min)-tightening of pump tubing	0.72, 0.84, 0.96	0.72, 0.84, 0.96	0.72, 0.84, 0.96	0.72, 0.84, 0.96
Flow rate of reducing agent and acid (mL/min)- pump diameter varying	0.85, 1.00, 1.23	0.85, 1.00, 1.23	0.85, 1.00, 1.23	0.85, 1.00, 1.23
Flow rate of sample (mL/min)-tightening of pump tubing	6.71, 7.05, 7.60	6.71, 7.05, 7.60	6.71, 7.05, 7.60	6.71, 7.05, 7.60
Flow rate of sample – pump diameter varying	6.50, 7.05, 7.52	6.50, 7.05, 7.52	6.50, 7.05, 7.52	6.50, 7.05, 7.52
Flow rate of acetylene(L/min)	2.5, 3.0, 3.5 and 4.0			Not applicable
Flow rate of argon (L/min)	0.2, 0.8, 1.2 and 2			
Delay time (s)	10, 20 and 30			45, 60, 70 and 90

The VGA 77 accessory is equipped with two flow rate adjustments one for the sample and the other for the reducing agent and acid. In order to regulate the flow rate, the adjustment knob was tightened or released to modify the flow rate.

To determine the flow rate for each solution the specific uptake tube for the respective solution (reducing agent, acid or sample) was placed in a 25 mL measuring cylinder and the volume of the reagent used in 60 s was determined to be the flow rate.

The pump tubing internal diameter was also varied to control the effect on flow rate. The internal diameter of the tubing used for the reducing agent and acid were 0.63 mm, 0.67 mm and 0.76 mm and for the sample 2.28 mm, 2.54 mm and 2.92 mm. The tubing for the sample was kept constant at 2.28 mm and the pump tubing for the acid and reductant were varied. The tubing size that produced the maximum signal for the acid and reductant was kept constant and the sample tubing varied.

The temperature of the air-acetylene flame was optimized for HG-AAS by regulating the flow rate (L/min) of the acetylene gas to oxidant. The acetylene and air flow rates were adjusted on the gas control interface of the Spectra AA software. The flow rate of argon used in the VGA 77 was adjusted from the flow gauge on the cylinder. The absorbance for the sample was recorded for each change in flow rate of the acetylene gas and argon. The instrument was zeroed after each adjustment was made. All conditions were kept constant during analysis only the parameter being optimized was varied.

The optimized conditions for each parameter were determined from those that produced the maximum sample absorbance.

## Discussion

**Fig. 2:**
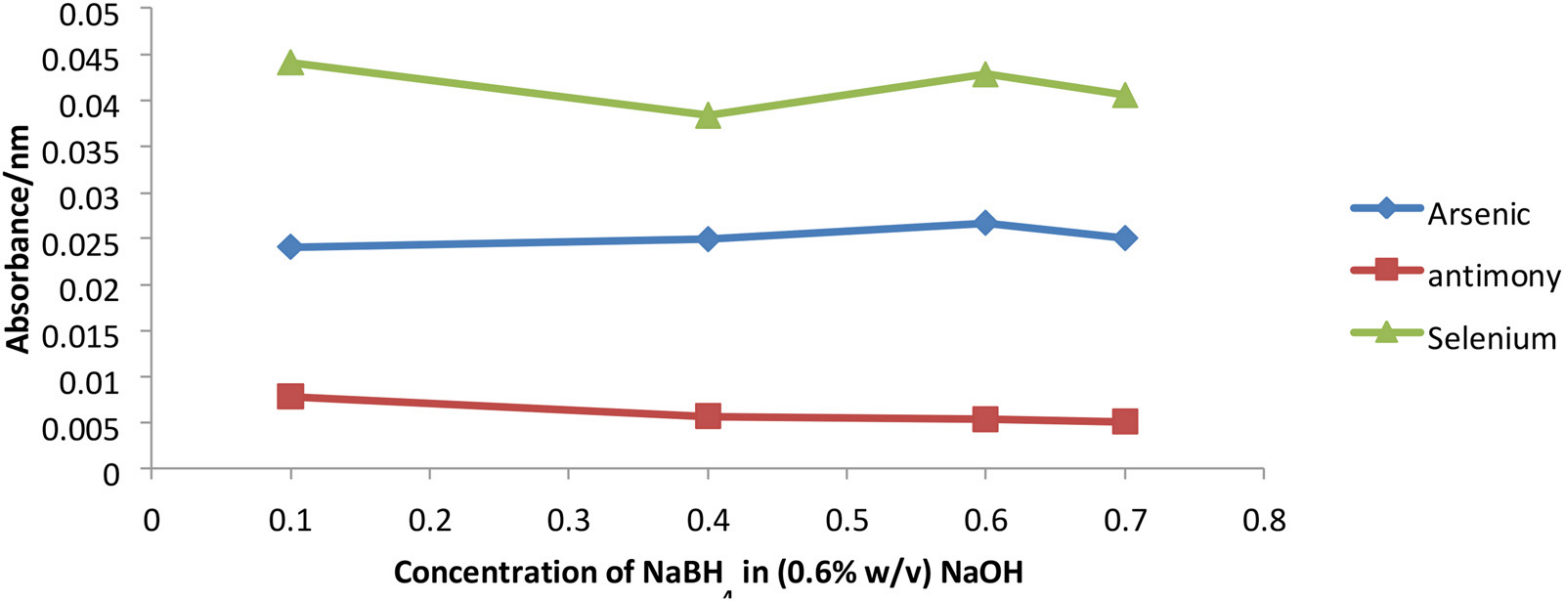
Graph of absorbance vs NaBH_4_ concentration for As, Sb and Se.

Optimized conditions are summarized in Table 2. The optimized instrumental conditions gave superior recoveries when used to measure volatile and hydride producing metals in the DORM-4 reference material as shown in Table 3. The reductant concentration had an important influence on signal intensity because it affects the hydride generation process, converting the oxidation state of the element to the more stable lower oxidation state. In the case of Mercury, Hg^2+^ is converted to Hg^0^, which forms the stable Mercury vapor. For Arsenic and Antimony, As^5+^ and Sb^5+^ are converted to As^3+^ and Sb^3+^, and for Selenium, Se^6+^ is reduced to Se^4+^. The reduced ions form the stable hydrides which are needed for HG-AAS. These reduced ions are produced by the reaction with an appropriate reducing agent. The optimum concentration of reducing agent used for Arsenic and Antimony was 0.6% (w/v) NaBH_4_ in 0.5% (w/v) NaOH (Fig. 2). For Selenium, the optimum reducing agent was 0.1% (w/v) NaBH4 in 0.5% (w/v) NaOH (Fig. 2) (Table 2).

**Table 2:** Optimized conditions for each parameter selected for each metal.

Parameter/Conditions	Arsenic	Antimony	Selenium	Mercury
Reducing agent concentration	NaBH_4_ (0.6% w/v) in 0.5% (w/v) NaOH	NaBH_4_ (0.6% w/v) in (0.5% w/v) NaOH	NaBH_4_ (0.1% w/v) in (0.5% w/v)NaOH	SnCl_2_ (25% w/v) in HCl (20% v/v)
Acid Concentration	5 M HCl	10 M HCl	10 M HCl	DI water
Flow rate of reducing agent and acid mL/min- pump adjustments	0.96 (max)	0.96 (max)	0.96 (max)	0.96 (max)
Flow rate of reducing agent and acid mL/min- varying pump tubing dimensions	1.00	1.00	1.00	1.00
Flow rate of sample (mL/min)	7.05	7.05	7.05	6.71
Flow rate of sample (mL/min)-pump tubing	7.05	7.00	7.05	6.50
Flow rate of argon (L/min)	0.2	0.2	2.0	2.0
Flow rate of acetylene (L/min)	2.5	2.5	2.5	NA
Delay Time (s)	20	20	20	70

**Table 3:** Showing recovery percentages for Dorm-4 Fish protein using the optimized and standard conditions.

Analyte	Percentage Recovery (Standard Conditions)	Percentage recovery (Optimized conditions)
Arsenic	87 %	91 %
Mercury	83 %	94 %
Selenium	85 %	96 %
Antimony	89 %	94 %

**Fig. 3:**
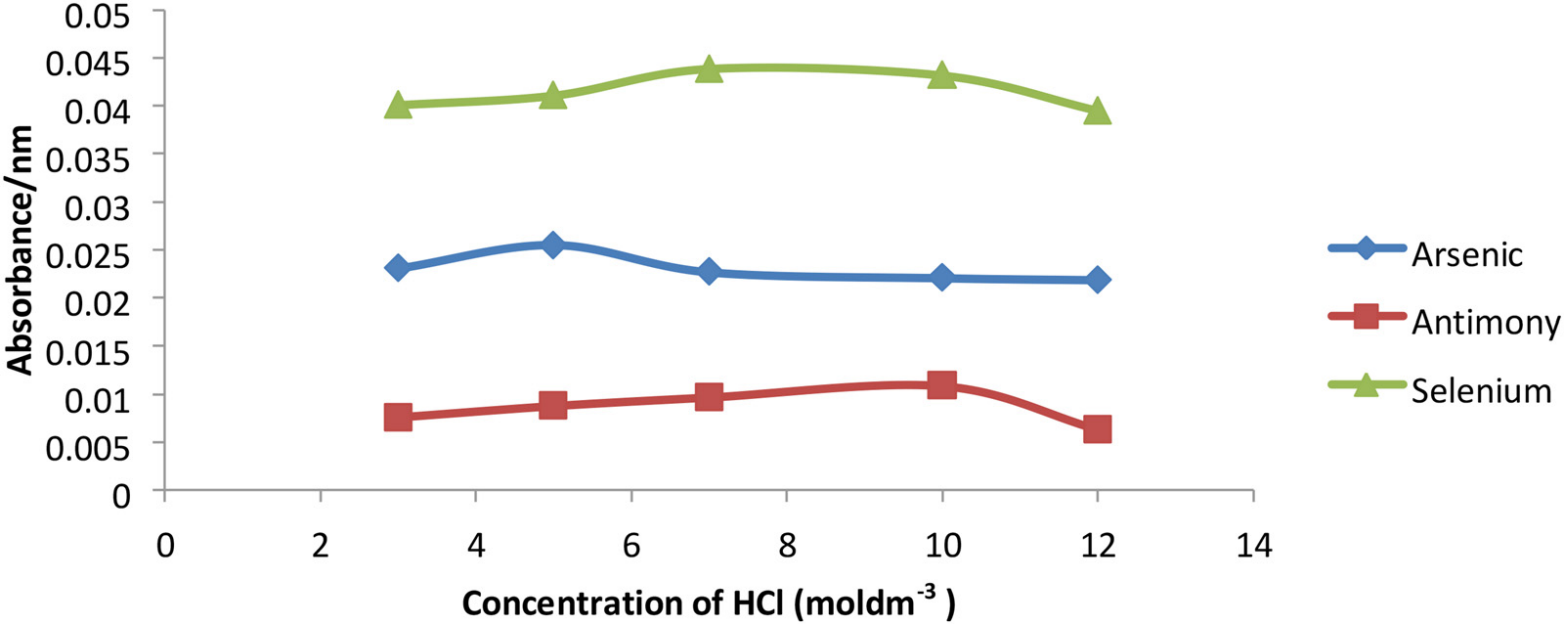
Graph of absorbance vs SnCl_2_ concentration for Hg.

The most efficient reducing agent for Mercury is well established to be SnCl_2_ [4]. The optimum concentration was found to be 25% (w/v) SnCl_2_ in 20% (v/v) HCl (Fig. 3). For Antimony and Mercury it was observed that using high concentrations of the reductant achieved better signal intensity, however for arsenic and selenium greater signal noise was produced with increasing concentrations of sodium borohydride [5].

The chemistry of vapor generation reactions indicate that at higher concentrations of acid reagent, the hydride generation processes should occur more rapidly. However elevated acid concentrations produces larger amounts of hydrogen gas which can dilute the hydride vapor thus reducing the concentration of the hydride [6].

**Fig. 4:**
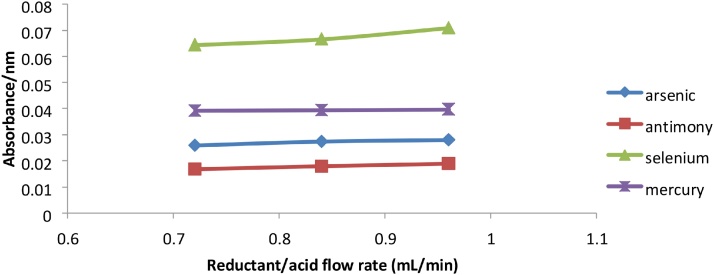
Graph of absorbance vs concentration of HCL for As, Sb and Se.

It is therefore essential to establish an optimal acid condition. The acid conditions for the hydride generating elements were as follows; 5 M HCl for Arsenic and 10 M HCl for Antimony and Selenium (Fig. 4).

**Fig. 5:**
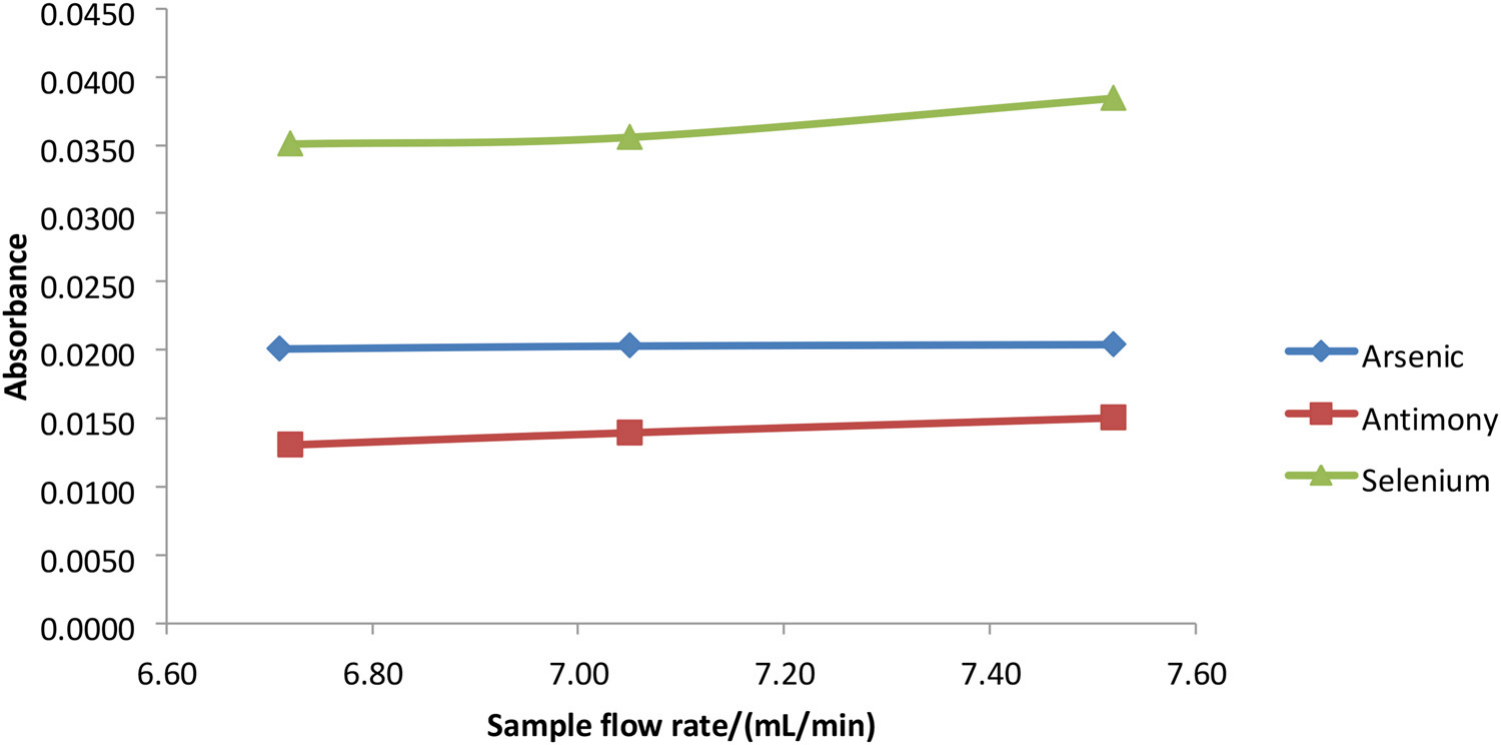
Graph of absorbance vs flow rate for reductant and acid for As, Sb, Se and Hg.

**Fig. 6:**
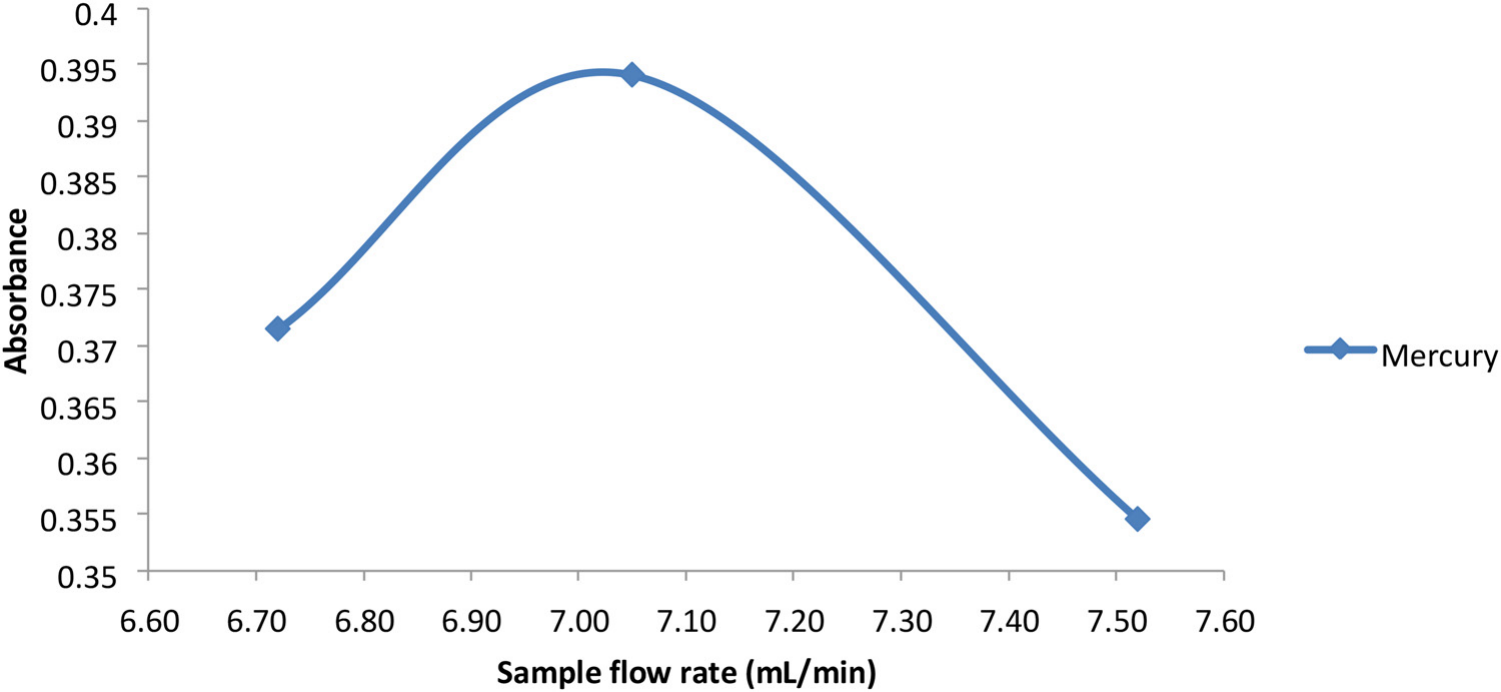
Graph of absorbance vs sample flow rate for As, Sb and Se.

**Fig. 7:**
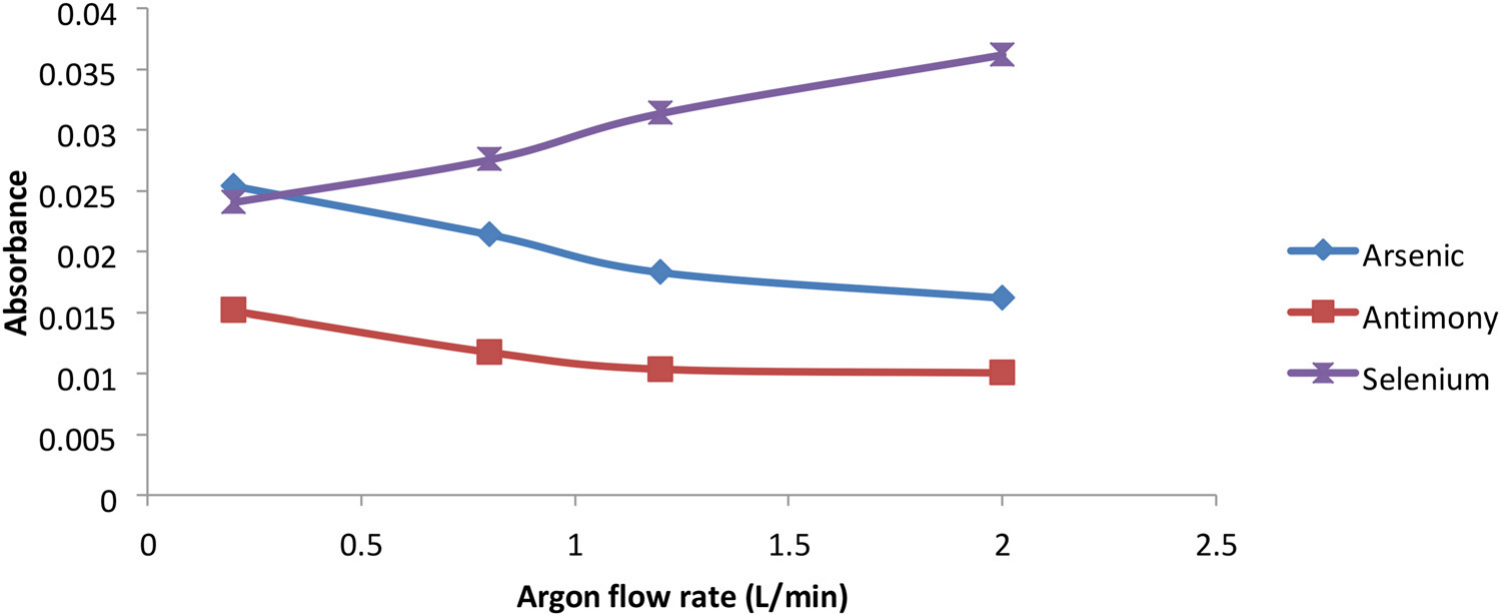
Graph of absorbance vs sample flow rate for Hg.

The flow rates of the reducing agent, acid and sample had an effect on sensitivity since the higher flow rates resulted in higher signal intensity. Too high a flow rate can result in a concentrated yet unstable vapor, and the ideal sample to reagent ratio is needed for maximum vapor generation. The optimum flow rate for reductant and acid for all the metals As, Sb, Se and Hg was 0.96 mL/min (Fig. 5), which was the maximum achievable flow on the VGA77. The maximum signal was obtained at a sample flow rate of approximately 7.0–7.05 mL/min for Arsenic, Antimony and Selenium while the maximum signal for Mercury was obtained at approximately 6.5–6.7 mL/min of sample (Fig. 6, Fig. 7).

**Fig. 8:**
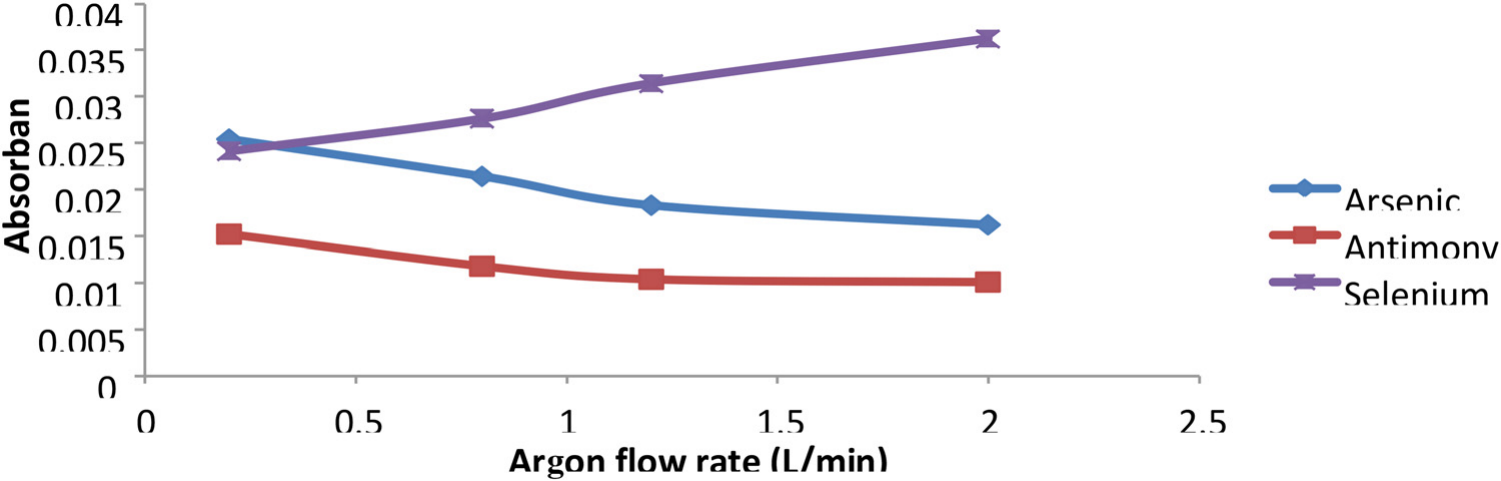
Graph of absorbance vs Argon flow rate for As, Sb and Se.

**Fig. 9:**
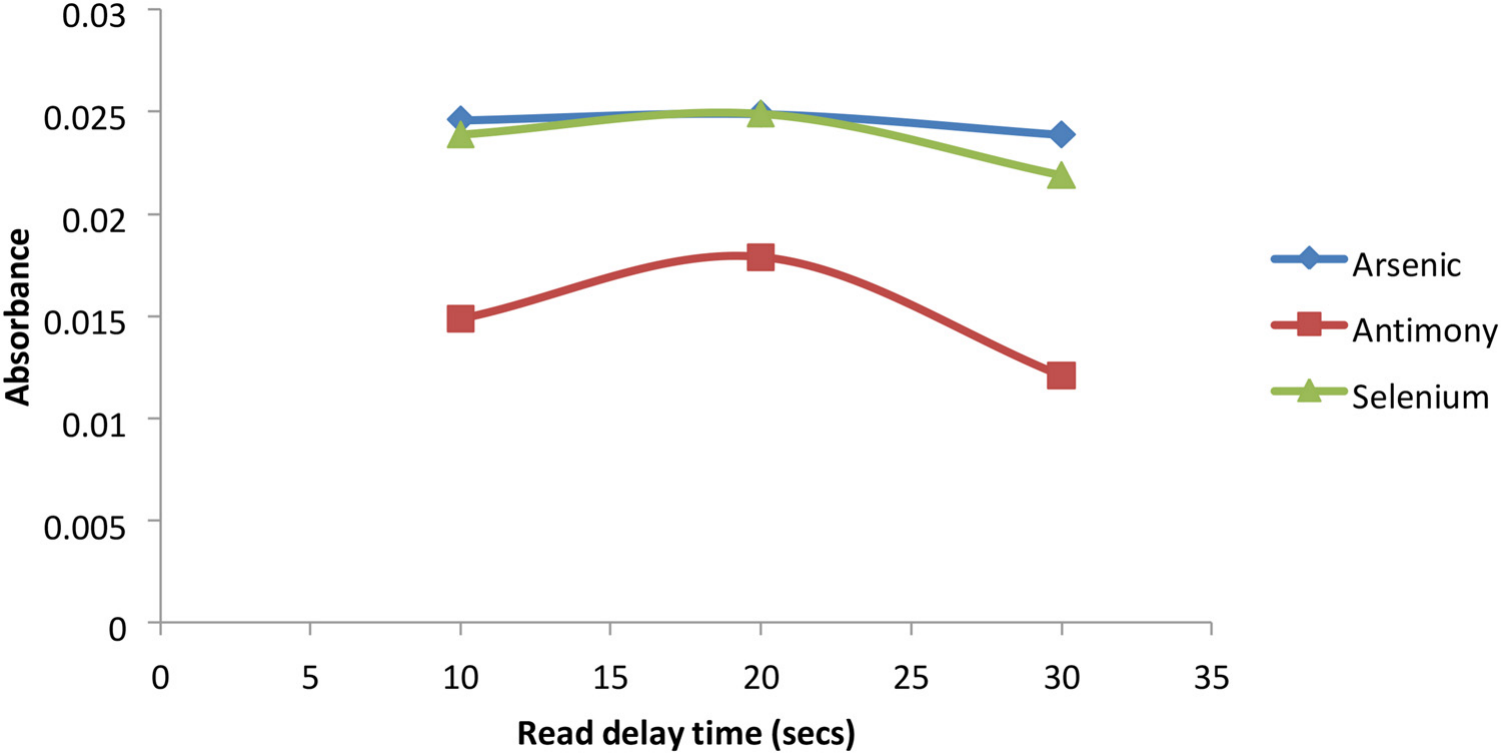
Graph of Absorbance vs Argon flow rate for Hg.

The Argon flow rate also played an important role in the hydride generation and cold vapor generation processes. As the carrier gas, the flow rate of the argon should be high enough to strip the volatile hydrides from the liquid mixtures and carry them up to the sample cell, but must not be so high so as to dilute the hydride formed. Mercury and Selenium produced higher signals with higher flow rates of Argon as compared to Arsenic and Antimony, where dilution of the hydride from excess Argon may have occurred. The optimum argon flow rates for Arsenic and Antimony was 0.2 mL/min while for Selenium and Mercury the optimum argon flow rate was 2.0 mL/min (Fig. 8, Fig. 9).

**Fig. 10:**
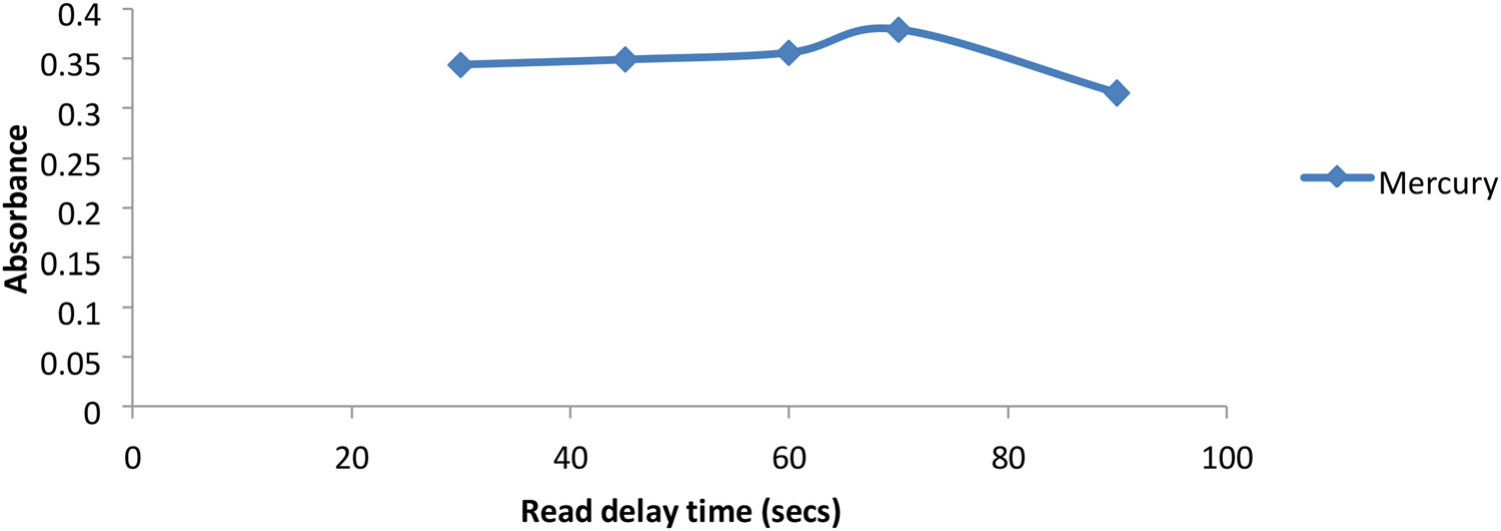
Graph of Absorbance vs read delay time (secs) for As, Sb and Se.

**Fig. 11:**
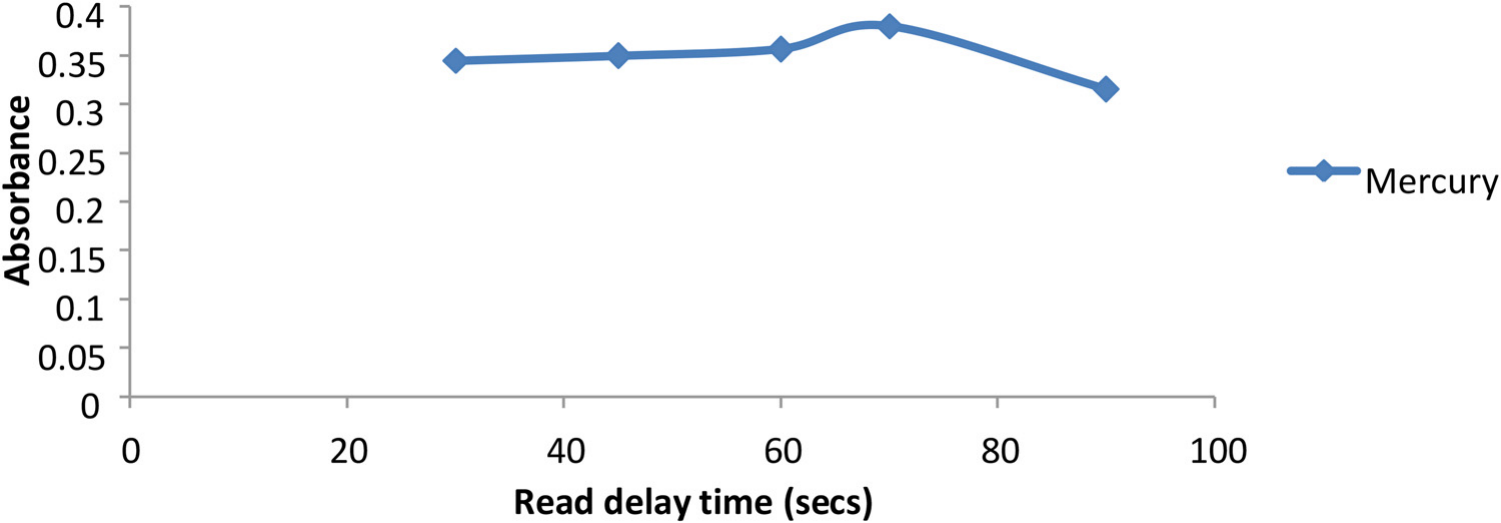
Graph of absorbance vs read delay time (sec) for Hg.

The VGA accessory uses chemical reactions to produce the elemental hydrides. The metal ions present in these hydrides can adhere to the surface of the various tubes that make up the VGA77, causing memory effects [7]. In order to effectively remove these adhered ions, an appropriate flushing time is needed between samples. This flushing time can be controlled by the instrument delay time. The longer the delay time, the more flushing can occur. The delay time was optimized to ensure that the possibility of memory effects was eliminated. Arsenic, Antimony and Selenium produced maximum signals at lower delay times of 20 s, however Mercury required longer delay times of up to 70 s to eliminate the memory effect (Fig. 10, Fig. 11).

## Supplementary material and/or additional information

NA.
